# Geospatial determinants and spatio-temporal variation of early initiation of breastfeeding and exclusive breastfeeding in Ethiopia from 2011 to 2019, a multiscale geographically weighted regression analysis

**DOI:** 10.1186/s12889-024-19552-0

**Published:** 2024-07-27

**Authors:** Tsion Mulat Tebeje, Beminate Lemma Seifu, Kusse Urmale Mare, Yordanos Sisay Asgedom, Zufan Alamrie Asmare, Hiwot Altaye Asebe, Abdu Hailu Shibeshi, Afework Alemu Lombebo, Kebede Gemeda Sabo, Bezawit Melak Fente, Bizunesh Fantahun Kase

**Affiliations:** 1 School of Public Health, College of Health Science and Medicine, Dilla University, Dilla, Ethiopia; 2 Department of Public Health, College of Medicine and Health Science, Samara University, Afar, Ethiopia; 3 Department of Nursing, College of Medicine and Health Sciences, Samara University, Afar, Ethiopia; 4 Department of Epidemiology and Biostatics, College of Health Sciences and Medicine, Wolaita Sodo University, Wolaita Sodo, Ethiopia; 5 Department of Ophthalmology, School of Medicine and Health Science, Debre Tabor University, Debre Tabor, Ethiopia; 6 Department of Statistics, College of Natural and Computational Science, Samara University, Afar, Ethiopia; 7 School of Medicine, College of Health Science and Medicine, Wolaita Sodo University, Wolaita Sodo, Ethiopia; 8 Department of General Midwifery, School of Midwifery, College of Medicine & Health Sciences, University of Gondar, Gondar, Ethiopia

**Keywords:** Early initiation of breastfeeding, Exclusive breastfeeding, Spatio-temporal analysis, EDHS, Geographically weighted regression, Multiscale geographically weighted regression, Ethiopia

## Abstract

**Background:**

Breastfeeding offers numerous benefits for infants, mothers, and the community, making it the best intervention for reducing infant mortality and morbidity. The World Health Organization (WHO) recommends initiating breastfeeding within one hour after birth and exclusively breastfeeding for the first six months. This study investigated the trend, spatio-temporal variation, and determinants of spatial clustering of early initiation of breastfeeding (EIBF) and exclusive breastfeeding (EBF) in Ethiopia from 2011 to 2019.

**Methods:**

Data from the Ethiopian Demographic and Health Survey (EDHS), which was conducted in 2011, 2016, and 2019, were analyzed utilizing a weighted sample of 10,616 children aged 0–23 years for EIBF and 2,881 children aged 0–5 months for EBF. Spatial autocorrelation analysis was used to measure whether EIBF and EBF were dispersed, clustered, or randomly distributed and Kriging interpolation was employed to predict the outcome variables in the unmeasured areas. Spatial scan statistics were used to identify spatial clusters with a high prevalence of cases. Both global and local regression modeling techniques were employed to examine the spatial relationships between the explanatory variables and the dependent variables.

**Results:**

The trend analysis revealed a notable increase in the prevalence of EIBF from 51.8% in 2011 to 71.9% in 2019. Similarly, the prevalence of EBF increased from 52.7% in 2011 to 58.9% in 2019. Spatial analysis demonstrated significant spatial variation in both EIBF and EBF throughout the country. Cold spots or clusters with a low prevalence of EIBF were observed consistently in the Tigray and Amhara regions, and significant cold spot areas of EBF were observed consistently in the Afar and Somali regions. Multiscale geographically weighted regression analysis revealed significant predictors of spatial variations in EIBF, including the religious affiliation of being a follower of the orthodox religion, parity of 1–2, absence of antenatal care visits, and delivery via cesarean section.

**Conclusions:**

Despite the increase in both EIBF and EBF rates over time in Ethiopia, these rates still fall below the national target. To address this issue, the government should prioritize public health programs aimed at improving maternal healthcare service utilization and maternal education. It is essential to integrate facility-level services with community-level services to achieve optimal breastfeeding practices. Specifically, efforts should be made to promote breastfeeding among mothers who have delivered via cesarean section. Additionally, there should be a focus on encouraging antenatal care service utilization and adapting maternal healthcare services to accommodate the mobile lifestyle of pastoralist communities. These steps will contribute to enhancing breastfeeding practices and achieving better outcomes for maternal and child health.

**Supplementary Information:**

The online version contains supplementary material available at 10.1186/s12889-024-19552-0.

## Introduction

Breast milk is the optimal source of nutrition for newborns, as it meets all nutritional needs for the first six months of life. Breastfeeding offers numerous benefits for infants, mothers and the community [1–3]. Infants benefit from a decreased incidence and severity of infectious diseases [4–6]; improved neurodevelopment [7, 8]; reduced risk of necrotizing enterocolitis [9]; and decreased risks of obesity, cardiovascular risk, diabetes [10] and post neonatal infant mortality [11]. Maternal health benefits include birth spacing [12], a quicker return to pre-pregnancy weight [13], reduced risk of breast cancer, ovarian cancer [14], cardiovascular diseases [15], type 2 diabetes [16] and maternal depression [17]. Breastfeeding plays a significant role in preventing the mortality of more than 820,000 children under 5 years of age and 20,000 deaths from breast cancer each year, contributing to the reduction in under-five and maternal mortality rates [18].

To obtain the benefits of breastfeeding, the WHO and the United Nations Children’s Fund (UNICEF) recommended initiating breastfeeding within one hour after birth and exclusively breastfeeding for the first six months [19]. However, despite efforts to promote early breast feeding initiation [20], only 47% of newborns globally begin breastfeeding within the first hour after birth [21]. In 2012, only 38% of infants under six months of age were exclusively breastfed, which increased to 40% in 2016 [22], and as of 2021, the rate has reached 48% [21]. This indicates that we are approaching the 2025 target for EBF, which is set at 50% [22]. Yet we still fall short of reaching the sustainable development goal (SDG) target for 2030, which is aimed at achieving a 70% EBF rate [23].

Between 2010 and 2018, the global weighted prevalence of early initiation of breastfeeding and exclusive breastfeeding in low- and middle-income countries (LMICs) was 51.9% and 45.7%, respectively [24]. In sub-Saharan Africa (SSA), the overall prevalence of EIBF was 52.8%, with the lowest prevalence observed in Guinea (16.5%) and the overall prevalence of EBF was 41.1%, with the lowest prevalence found in Gabon (6.04%) [25].

Regardless of the progress that has been achieved in Ethiopia, mainly due to different initiatives and programs (such as infant and young child feeding practices, training of health-extension workers and baby-friendly hospital initiatives [26, 27]), only 61.4% of neonates are breastfed within an hour after delivery and just 59.3% of infants are breastfed exclusively [28, 29]. The country is still falling behind in achieving SDG and its national target which is to increase the proportion of EIBF to 92% and that of EBF to 70% [30]. Previously conducted research in Ethiopia have identified factors that are associated with EIBF and EBF, including religion, region, area of residence, household wealth index, marital status, media exposure, maternal education, maternal age, employment status of the mother, antenatal care, place of delivery, mode of delivery, postnatal care, sex of the child and parity [31–35].

Studies have reported significant geographical variation in the prevalence of EIBF and EBF across regions in Ethiopia [35] and one study focused solely on the trends in EIBF and EBF without demonstrating how these trends aligned with geographical variations [34]. To address these limitations, we conducted a comprehensive trends and spatio-temporal analyses of EIBF and EBF from 2011 to 2019. Furthermore, our study incorporated a multiscale geographically weighted regression (MGWR) analysis to overcome the limitations of another prior study [36] that relied on geographically weighted regression (GWR) to investigate the relationships between the EIBF and independent variables. The MGWR model examines the local spatial relationships between clusters of EIBF and EBF and their predictors. This approach improves upon the drawbacks of GWR by adjusting both the space and scale, allowing variables to operate at different scales [37]. Identification of significantly varying predictors across space helps to implement interventions that increase location-specific governmental effort in improving the magnitudes of EIBF and EBF and achieving targets. Hence, the objective of this study was to investigate the trend, spatial variation and spatial determinants of EIBF among children aged 0–23 months and EBF among infants aged 0–5 months in Ethiopia.

## Methods and materials

### Study setting, design and data

The study was conducted in Ethiopia, which is located in the Horn of Africa. The country is divided into 11 regions and two city administrations, including the two regions which were introduced after the surveys were conducted. Tigray, Afar, Amhara, Oromia, Southern Nations Nationalities and People (SNNP), Benishangul Gumuz, Gambella, Somali, Harari, Sidama, Southwest Ethiopia, Addis Ababa and Dire Dawa [38].

The data used in this study were obtained from three consecutive nationally representative cross-sectional surveys: EDHS 2011 [39], 2016 [40] and 2019 [41]. All the EDHS used a two-stage stratified cluster sampling procedure involving the selection of clusters in the first stage and the selection of households in the second stage. Cluster selection was stratified by place of residence (urban or rural area) and district or woreda, which was further subdivided into kebeles, and each kebele was then subdivided into enumeration areas (EAs). Location data or latitudinal and longitudinal coordinates were included in the selected EAs.

### Population

The source populations for this study were all children aged 0–23 months and all children aged 0–5 months in Ethiopia for EIBF and EBF, respectively. We used the Kids record dataset (KR file), and a total weighted sample of 10,616 children aged 0–23 months was included for the EIBF 2011 (4,308), 2016 (4,222) and 2019 (2,086) surveys. A total of 2,881 weighted children aged 0–5 months were analyzed for the EBF 2011 (1179), 2016 (1162) and 2019 (540) surveys after excluding children who did not live with their mother and keeping only the youngest child.

### Study variables

The dependent variables for this study were early initiation of breastfeeding (yes/no) and exclusive breastfeeding (yes/no). EIBF was defined as the percentage of children aged 0–23 months who were put to the breast within one hour of birth. EBF was defined as the percentage of infants aged 0–5 months who were fed exclusively breast milk with no other food or drink, not even water [42]. The independent variables considered in the study were residence, household wealth index, marital status, media exposure, maternal education, maternal age, employment status of the mother, antenatal care, place of delivery, mode of delivery, postnatal care, sex of the child and parity. The geographical regions of the participants were categorized into three regions: the larger central region (Tigray, Amhara, Oromia, and SNNP), the small peripheral region (Afar, Somali, Benishangul, and Gambella) and the metropolis region (Harari, Dire Dawa, and Addis Ababa) [43]. Participants were considered to have media exposure if they watched television, listened to the radio or read a newspaper [44].

### Data management and analysis

Data extraction, coding and descriptive analysis were conducted using Stata version 17. The data were weighted using sampling weights before any statistical analysis to make them representative. We employed frequency and percentage distributions to report respondent characteristics and trends. The weighted proportions of the dependent and independent variables were extracted by cross tabulating with the cluster number (v001) and exported as a CSV file. The data were subsequently exported to ArcGIS version 10.7 for exploration and visualization of EIBF and EBF in Ethiopia at the regional level during the three survey years. Spatial regression analysis and visualization of significant variables were conducted with Python 3 software.

#### Spatial autocorrelation

Spatial autocorrelation was employed to examine whether the spatial patterns of EIBF and EBF in Ethiopia were randomly distributed, dispersed or clustered. Global Moran’s I statistics were used to identify the spatial pattern. The range of Moran’s I is from − 1 to 1. A value close to -1 indicates strong dispersion, a value close to 1 indicates strong clustering, and a value close to 0 indicates no spatial autocorrelation or randomness. A statistically significant Moran’s I value reflects the presence of spatial autocorrelation [45, 46].

#### Hotspot analysis

Hotspot analysis was also conducted to identify areas with statistically significant clustering. The Getis–Ord Gi* statistic was calculated to determine the presence of concentrations that exhibited significantly higher or lower values. It generates an output feature class containing the z score, *p* value and confidence level bin field (Gi_Bin). The Gi_Bin field categorizes areas of EIBF and EBF into statistically significant hot spots and cold spots at different confidence levels (99%, 95% and 90%). A feature is considered a statistically significant hotspot if it has a high value and is surrounded by other features with high values. On the other hand, a feature is deemed a statistically significant cold spot if it has a low value and is surrounded by other features with low values [47, 48].

#### Spatial interpolation

A spatial interpolation technique was used to predict the spatial patterns of EIBF and EBF in areas where data were not directly observed from the sampled measurements. The kriging spatial interpolation method was utilized to make predictions about unsampled areas [49].

#### Spatial scan analysis

Spatial scan statistics was conducted to detect statistically significant spatial clusters with high and low prevalence of EIBF and EBF in Ethiopia. A circular scanning window was used to traverse the study area. The Bernoulli-based model was employed using Kuldorff’s SaTScan version 9.6 software. The case (presence of an outcome variable), control (absence of an outcome variable) and coordinate file (latitude and longitude) were taken to the software to determine the location of the significant clusters. Most likely or primary clusters were identified using *p* values and likelihood ratio tests. The cluster with the maximum likelihood ratio constitutes the primary cluster [50]. Using the default maximum spatial cluster size of 50% in terms of the total population at risk could hide small core clusters, and a too small maximum size could miss significant clusters in a larger size [51]. Hence, we set the maximum spatial cluster size to 20% of the population at risk to ensure the resulting circles were of a modest size.

### Modeling spatial relationships

Using the most recent survey data from the EDHS 2019, we employed spatial regression analysis to identify predictors of the observed spatial patterns of EIBF and EBF in Ethiopia. To examine the spatial relationship between the explanatory variables and the dependent variables, both global and local regression modeling approaches were employed.

### Global regression modeling

The ordinary least square (OLS) regression analysis is a global model that was applied as a preliminary test of the correlation between the dependent and independent variables [52]. It assumes a stationary and constant relationship over space, which implies that the relationships do not vary over space [53]. To assess the magnitude of multicollinearity in the model, the variance inflation factor (VIF) was utilized. The OLS model is calculated as follows:

$${y}_{i}={\beta\:}_{0}+\sum\beta_{i}{x}_{i}+{\varepsilon}_{i}$$

where yi is the dependent variable, β0 is the intercept, xi represents the independent variable, βi is the corresponding coefficient and ε is the error [52].

### Local regression modeling

Geographically weighted regression relaxes the assumption of OLS regression by allowing the coefficients to vary spatially. GWR is employed when the Koenker statistics is significant, indicating that the relationships between the outcome and the covariates change from location to location. GWR assumes that the relationships between the outcome and explanatory variables are nonstationary, meaning that they vary spatially. Therefore, the GWR model estimates a local parameter for each location separately. However, it assumes that all the coefficients change at a similar rate across the study area and uses a single constant bandwidth [54]. The GWR model is calculated as follows:

$${y}_{i}={\beta}_{0\left(ui,vi\right)}+\sum\limits_{k}{\beta}_{k\left(ui,vi\right)}{x}_{ik}+{\varepsilon}_{i}$$

where (*ui*,* vi*) represents coordinates; *β0* and *βk* are the intercept and the coefficient of local variable *k* at location *i*, respectively; and *xik* is the kth variable at location *i* [52].

The multiscale geographically weighted regression model is an extended and advanced version of GWR. This approach allows the relationship between dependent and independent variables to vary spatially and at different spatial scales by using different bandwidths rather than a single constant bandwidth across the study area [53, 55]. The MGWR model is calculated as follows:

$${y}_{i}={\beta}_{{bw}_{0}(ui,vi)}+\sum\limits_{k}{\beta}_{{bw}_{k}\left(ui,vi\right)}{x}_{ik}+{\varepsilon}_{i}$$

The parameters are almost the same as those of GWR, except for the indicated label bw, which represents the different bandwidths of each variable [52].

We assessed the presence of multicollinearity using the variance inflation factor (VIF). The performances of both the local and global models, were compared by the Akaike information criterion (AIC), residual sum of squares (RSS), and adjusted R-squared values [56].

## Results

### Trends in EIBF and EBF in Ethiopia

We included a total weighted sample of 10,616 children aged 0–23 months and 2,881 children aged 0–5 months in the analysis. The majority of the children were from the larger central regions, while metropolitan areas contained the minority of children in both age groups. Table 1 shows the weighted frequency of participant characteristics in each survey year, with the proportion of EIBF and EBF in parentheses.

**Table 1:** Trends in EIBF and EBF by characteristics in 2011, 2016 and 2019 Ethiopian demographic and health survey

Characteristics	Variables	Weighted frequency (% EIBF)			Weighted frequency (% EBF)		
		2011	2016	2019	2011	2016	2019
**Total**		**4308 (51.8)**	**4222 (73.6)**	**2086 (71.9)**	**1179 (52.7)**	**1162 (57.3)**	**540 (58.9)**
Residence	Urban	581 (57.2)	499 (73.9)	551 (70.3)	133 (49.1)	135 (58.1)	124 (53.4)
	Rural	3727 (51.0)	3723 (73.5)	1535 (72.4)	1046 (53.1)	1027 (57.1)	415 (60.6)
Religion	Orthodox	1604 (44.5)	1425 (65.4)	740 (65.6)	398 (67.1)	376 (66.6)	180 (65.3)
	Muslim	1543 (50.6)	1775 (77.7)	757 (75.9)	480 (39.9)	529 (51.5)	229 (51.3)
	Protestant	1024 (64.1)	866 (76.0)	550 (75.1)	262 (56.6)	207 (53.9)	129 (64.2)
	Others	138 (61.7)	155 (87.1)	39 (64.9)	39 (37.0)	50 (60.6)	2 (11.5)
Wealth index	Poor	1974 (49.9)	1934 (75.0)	904 (73.2)	546 (52.1)	561 (59.0)	258 (59.8)
	Middle	895 (51.0)	874 (73.5)	389 (70.2)	269 (58.1)	215 (54.6)	100 (73.7)
	Rich	1439 (54.9)	1414 (71.7)	793 (71.1)	364 (49.6)	386 (56.2)	181 (49.6)
Marital status	Single	487 (50.3)	252 (65.9)	117 (71.2)	136 (58.4)	54 (56.5)	29 (75.4)
	Married	3820 (52.0)	3970 (74.1)	1969 (71.9)	1043 (51.9)	1107 (57.3)	510 (58.0)
Media exposure	Not exposed	2561 (50.2)	2879 (73.5)	1340 (71.4)	690 (54.5)	810 (56.5)	348 (59.8)
	Exposed	1747 (54.2)	1343 (73.7)	746 (72.5)	488 (50.1)	351 (58.9)	192 (57.4)
Maternal age	15–19	297 (40.9)	272 (66.1)	179 (64.3)	91 (48.0)	105 (51.0)	51 (48.7)
	20–24	984 (53.8)	948 (74.7)	494 (73.4)	289 (48.2)	278 (59.0)	114 (70.9)
	25–29	1380 (50.4)	1238 (75.3)	637 (72.0)	354 (53.3)	326 (57.8)	137 (67.5)
	30–34	776 (53.2)	913 (76.3)	391 (68.6)	199 (59.0)	238 (57.5)	131 (43.9)
	35–39	580 (57.8)	586 (68.4)	260 (75.2)	181 (52.7)	164 (56.2)	71 (71.1)
	40–44	221 (44.6)	205 (71.6)	112 (78.7)	58 (56.7)	41 (57.6)	34 (34.0)
	45–49	69 (56.8)	60 (69.6)	14 (86.7)	7 (54.2)	9 (66.9)	3 (0.0)
Literacy	Illiterate	3291 (50.8)	3064 (73.8)	1226 (72.3)	876 (54.6)	828 (57.6)	339 (58.0)
	Literate	1012 (55.4)	1158 (73.0)	859 (71.2)	299 (47.5)	333 (56.5)	200 (60.5)
Education level	No education	2890 (50.8)	2588 (73.4)	974 (71.4)	779 (54.9)	694 (57.4)	279 (59.8)
	Primary	1231 (52.0)	1280 (74.2)	834 (72.8)	342 (47.8)	353 (58.3)	189 (59.0)
	Secondary and above	187 (66.2)	354 (72.4)	278 (70.7)	58 (51.8)	115 (53.4)	71 (55.1)
Antenatal care	No	2444 (49.9)	1498 (74.5)	543 (71.4)	651 (54.6)	407 (53.0)	161 (62.5)
	01-Mar	1114 (52.6)	1311(73.3)	621 (72.6)	339 (52.8)	394 (60.3)	151 (55.7)
	4+	750 (56.8)	1414 (72.8)	922 (71.6)	190 (45.8)	361 (58.7)	226 (59.0)
Place of delivery	Home	3782 (51.8)	2631 (73.6)	939 (70.1)	1041 (53.2)	725 (56.5)	257 (62.5)
	Health facility	526 (52.1)	1591 (73.6)	1146 (73.3)	138 (48.8)	437 (58.6)	282 (55.7)
Mode of delivery	Vaginal	4221 (52.2)	4104 (74.6)	1956 (73.3)	1159 (53.0)	1128 (57.5)	507 (60.9)
	Cesarean section	87 (32.8)	118 (37.7)	129 (49.6)	20 (33.2)	34 (48.1)	33 (28.2)
Postnatal checkup	No	3902 (51.7)	3951 (74.0)	1887 (72.9)	1077 (53.4)	1090 (58.7)	488 (59.5)
	Yes	132 (60.3)	271 (67.0)	199 (62.5)	33 (39.0)	72 (35.0)	50 (55.3)
Sex of the child	Male	2246 (49.4)	2055 (71.6)	1076 (70.6)	628 (50.1)	568 (56.3)	255 (58.2)
	Female	2062 (54.4)	2167 (75.4)	1010 (73.2)	551 (55.6)	594 (58.2)	284 (59.6)
Parity	1–2	1471 (50.3)	1516 (71.7)	916 (68.6)	400 (48.5)	442 (56.1)	206 (62.6)
	3–4	1206 (51.3)	1112 (74.4)	523 (70.5)	315 (56.5)	307 (61.2)	139 (54.3)
	5–6	800 (54.0)	791 (74.9)	370 (74.7)	217 (60.6)	193 (59.6)	105 (60.7)
	7+	831 (53.2)	804 (74.5)	277 (81.5)	247 (47.6)	220 (51.9)	90 (55.7)
Region	Larger central	3962 (51.9)	3814 (73.8)	1808 (72.9)	1077 (55.0)	1050 (58.1)	469 (61.9)
	Small peripheral	228 (44.7)	274 (71.2)	197 (61.9)	74 (24.5)	80 (45.8)	53 (34.9)
	Metropolis	118 (51.8)	133 (73.3)	80 (72.2)	28 (37.0)	32 (57.7)	17 (53.4)

The prevalence of early initiation of breastfeeding in Ethiopia increased from 51.8% (95% CI: 50.3%, 53.3%) in 2011 to 73.6% (95% CI: 72.2%, 74.9%) in 2016 and decreased to 71.9% (95% CI: 69.9%, 73.7%) in 2019. Similarly, the prevalence of exclusive breastfeeding increased from 52.7% (95% CI: 49.8%, 55.5%) in 2011 to 57.3% (95% CI: 54.4%, 60.0%) in 2016 to 58.9% (95% CI: 54.7%, 63.0%) in 2019. The small peripheral regions (Afar, Somali, Benushangul and Gambela) had the lowest prevalence of both EIBF and EBF across all survey years, while the larger central regions had the highest prevalence. (Table 1)

### Spatial analysis

#### Spatial variation in EIBF and EBF in Ethiopia

Spatial autocorrelation revealed significant spatial variations in EIBF (Supplementary Fig. 1) and EBF (Supplementary Fig. 2) throughout Ethiopia in all three surveys conducted from 2011 to 2019. The global Moran’s I values revealed a significantly clustered pattern for both outcomes, with a *p*-value less than 0.05. These findings suggested that the clustering pattern is less likely to be a result of random chance. Rather, both EIBF and EBF exhibited significantly clustered patterns. This finding implies the need to investigate the two outcomes across different regions of the country.

#### Hotspot analysis of EIBF and EBF in Ethiopia

**Fig. 1:**
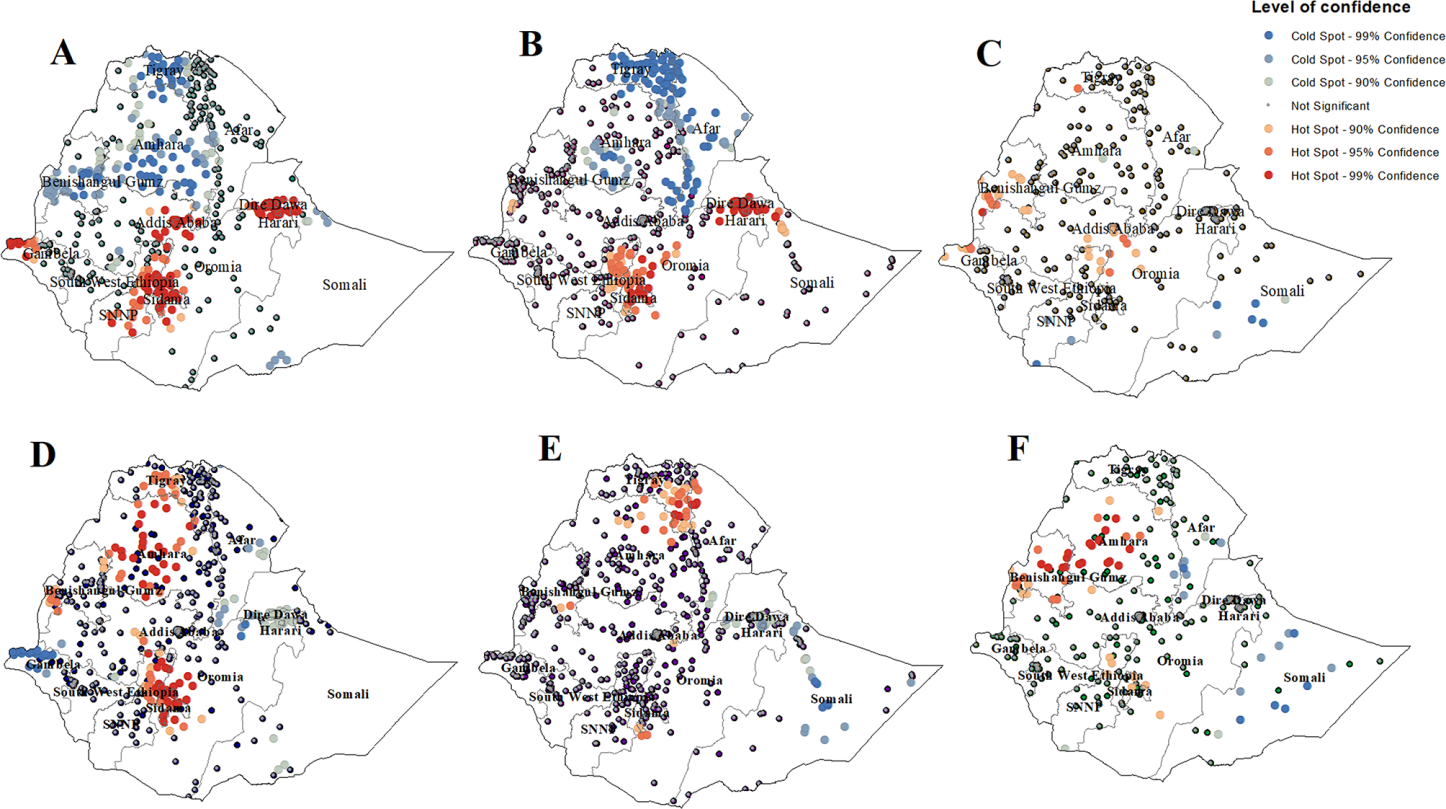
Hotspot analysis of EIBF among children aged 0–23 months in 2011 (**A**), 2016 (**B**), and 2019 (**C**) and EBF among children aged 0–5 months in 2011 (**D**), 2016 (**E**), and 2019 (**F**) in Ethiopia

In the 2011 EDHS, hotspots (high numbers of cases of EIBF surrounded by high numbers of cases) among children younger than 24 months were identified in various regions, including Dire Dawa, Harari, Addis Ababa, parts of eastern and central Oromia, northern Gambela, Sidama and eastern and central SNNP. Conversely, significant cold spot (low numbers of cases of EIBF are surrounded by low cases) areas were observed in central Tigray; central, southern and some parts of northern Amhara; and central, eastern and western Benishangul regions (Fig. 1A). By the 2016 EDHS, hotspots of EIBF were still spotted in Dire Dawa, Harari, parts of eastern and central Oromia, Sidama and northern SNNP. Cold spot areas were detected in the central and eastern Tigray, central and southern Afar, and southern and central Amhara regions (Fig. 1B). Moving to the 2019 EDHS, EIBF hotspots were concentrated in Addis Ababa, some parts of central Oromia, parts of northern SNNP, some parts of northern Gambela and southern and western Benishangul. Cold spots of EIBF were identified in southern parts of the SNNP and certain areas of the central Somali region (Fig. 1C).

In terms of EBF, in the 2011 EDHS hotspot areas of EBF, where high cases of EBF were surrounded by high cases, were detected in central Tigray, northern and central Amhara, western Benishangul, northern SNNP, Sidama and some parts of central Oromia. On the other hand, cold spots of EBF were identified in northern Gambela, some parts of central and southern Afar, and some parts of the northeastern Oromia region (Fig. 1D). Moving to the 2016 EDHS, hotspots of EBF were identified in eastern and southern Tigray, the western border of Afar with Tigray, northeastern Amhara, a part of southeastern Benishangul and a part of southern Oromia at the border with the Gedeo zone in the SNNP. Cold spot areas were observed in the central and eastern Somali region (Fig. 1E). Finally, in the 2019 EDHS, EBF hotspots were identified in central and northern Amhara, most parts of Benishangul, northern SNNP and the Sidama region. Cold spots were detected in central and eastern Somali, central Afar and southern Afar regions bordering the Amhara region (Fig. 1F).

#### Spatial interpolation of EIBF and EBF in Ethiopia

**Fig. 2:**
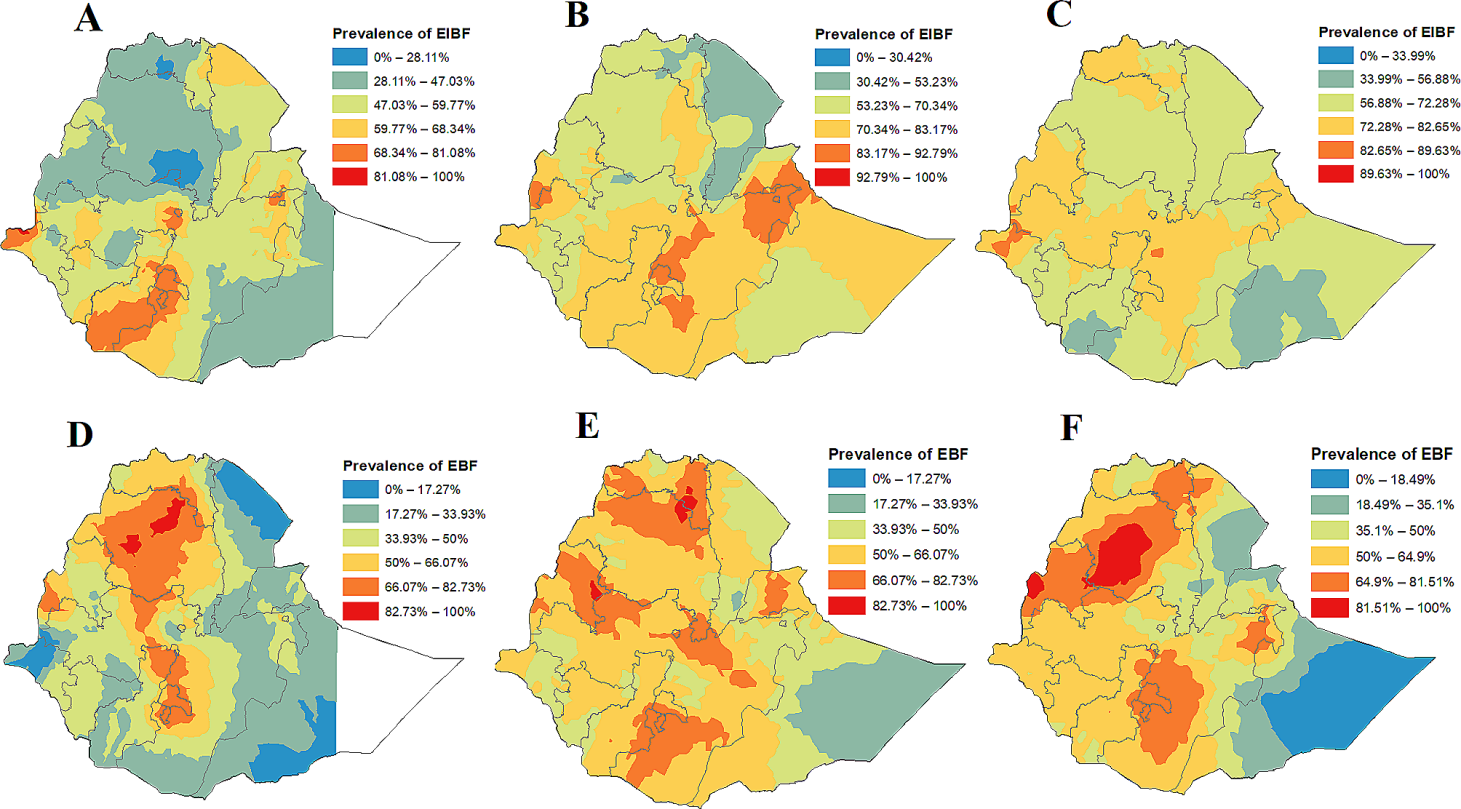
Spatial interpolation of EIBF among children aged 0–23 months in 2011 (**A**), 2016 (**B**), and 2019 (**C**) and EBF among children aged 0–5 months in 2011 (**D**), 2016 (**E**), and 2019 (**F**) in Ethiopia

We used kriging interpolation to predict geographical variations in EIBF and EBF in unpredicted areas based on the observed data. The resulting predictions were represented using a color scheme in which green indicates predicted areas with lower proportions of EIBF and EBF, while red indicates areas with higher proportions of EIBF and EBF. According to the sampled data from the 2011 EDHS, interpolation predicted the highest prevalence of EIBF in Addis Ababa, Dire Dawa, northern Gambela, Sidama and southern and eastern SNNP (Fig. 2A). Similarly, in the 2016 EDHS, the highest prevalence of EIBF was predicted in Dire Dawa, Harari, parts of eastern, central and southern Oromia, parts of northern Somali and some parts of southern Benishangul based on the sampled data (Fig. 2B). Similarly, for the 2019 EDHS, the predicted highest prevalence of EIBF was concentrated in parts of northwestern Gambella and the southwestern part of the Benishangul borderline with Oromia (Fig. 2C) based on the sampled areas.

In the 2011 EDHS, the highest prevalence of EBF was predicted based on sampled areas in the northern, central and western parts of Amhara, parts of southwestern Benishangul, northern SNNP, Sidama provinces and parts of northern and southwestern Oromia (Fig. 2D). According to the 2016 EDHS, the highest prevalence of EBF was predicted in various regions, including western, eastern and southern Tigray, northern and northeastern Amhara, central and eastern Benishangul, Addis Ababa, central and southern Oromia, Sidama and some parts of the eastern SNNP and northern Somali (Fig. 2E). Moving to the 2019 EDHS, the sampled data predicted the highest prevalence of EBF in central, western and northeastern Amhara; most parts of Benishangul; parts of central southern and eastern Oromia; some areas in the eastern SNNP bordering Sidama and Oromia; and the Sidama region (Fig. 2F).

#### Sat-scan analysis

**Fig. 3:**
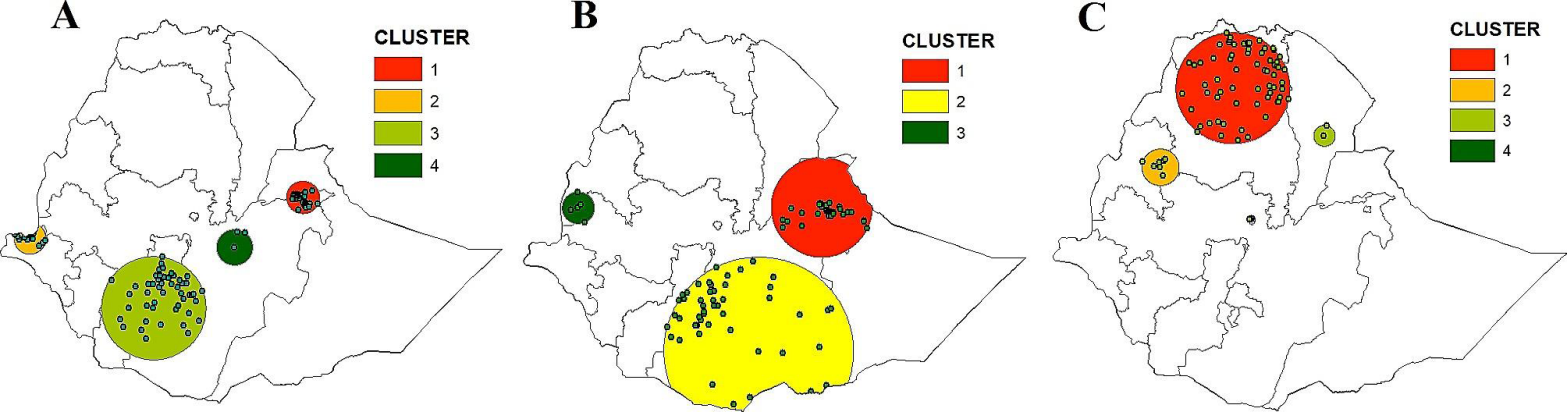
SaTScan analysis of EIBF among children aged 0–23 months 2011 (**A**), 2016 (**B**) and EBF among children aged 0–5 months 2016 (**C**) in Ethiopia

The results of the SaTScan analysis of EIBF and EBF throughout the EDHS survey years are displayed in Fig. 3. The most likely primary and secondary clusters of EIBF and EBF were identified. For EIBF, a total of 129 locations for the primary and secondary clusters were detected in 2011, 103 in 2016 and 39 in 2019. Similarly, for EBF, a total of 30, 84 and 11 primary and secondary clusters were detected in 2011, 2016 and 2019, respectively. (Table 2)

**Table 2:** SaTScan analysis results for EIBF and EBF among children aged 0–23 months and 0–5 months, respectively, in Ethiopia (2011–2019)

Cluster	EDHS year		Number of locations	Population	Case	Prevalence	RR	LLR	*P*-value
		**EIBF**							
1	EDHS 2011		65	475	313	0.66	1.24	17.7	< 0.001
2			13	117	91	0.77	1.48	15.5	< 0.001
3			48	471	304	0.64	1.25	13.9	< 0.001
4			3	32	25	0.78	1.47	4.29	0.982
1	EDHS 2016		50	400	322	0.80	1.29	27.6	< 0.001
2			48	440	337	0.77	1.22	17.1	< 0.001
3			5	49	40	0.82	1.27	3.52	0.996
1	EDHS 2019		13	123	98	0.79	1.23	6.17	0.260
2			20	171	130	0.76	1.18	4.68	0.708
3			6	66	54	0.82	1.26	4.36	0.865
		**EBF**							
1	EDHS 2011		30	88	53	0.60	1.30	2.97	0.96
1	EDHS 2016		69	148	111	0.75	1.42	13.3	0.0011
2			7	18	18	1.00	1.81	10.57	0.011
3			2	6	6	1.00	1.79	3.49	0.998
4			6	6	6	1.00	1.79	3.49	0.998
1	EDHS 2019		11	49	32	0.65	1.37	2.68	1.00

In the EDHS 2011, the primary cluster spatial window of EIBF was found in Dire Dawa and Harari, with a relative risk (RR) of 1.24 and a log likelihood ratio (LLR) of 17.7, at a *p*-value < 0.001. It revealed children between the ages of 0 and 23 months in the spatial window had a 1.28-fold greater chance of having early initiation of breastfeeding than did those outside the window (Fig. 3A). Based on the 2016 EDHS, the primary or most likely cluster was identified in Eastern Oromia, northern Somali, Harari and Dire Dawa (Fig. 3B), with an RR of 1.29 and an LLR of 27.6, at a *p*-value < 0.001. This showed that children under the age of 24 months in this spatial window had a 1.3 times greater chance of experiencing EIBF than children outside the window. According to the 2019 EDHS, the spatial window of the primary spatial cluster resulted in an RR of 1.23, an LLR of 6.17 and a *p*-value of 0.26. This clustering is more likely to occur as a result of chance because the nonsignificant *p*-value prevents us from ruling out that the cluster was formed by chance. (Table 2)

Considering exclusive breastfeeding, in the 2011 and 2019 EDHS, the spatial window of the primary clusters resulted in RR of 1.30 and 1.37, LLR of 2.97 and 2.68, and *p*-value of 0.96 and 1.00, respectively. This revealed that clustering was more likely to occur by chance in both survey years, as the nonsignificant *p*-values failed to rule out that the clusters were formed by chance. The spatial window of the primary cluster in the 2016 EDHS was located in most parts of Tigray and central and northern Amhara, with an RR of 1.42, an LLR of 13.3 and a *p*-value of 0.0011. The significant *p*-value ruled out the possibility that the cluster was formed by chance. Therefore, children between the ages of 0 and 5 months are 1.42 times more likely to have EBF than are those outside the window (Fig. 3C) (Table 2).

### Spatial regression analysis

We conducted an ordinary least square regression to investigate the assumptions of spatial regression. The model for EIBF yielded statistically significant results, as indicated by the significant Joint F-statistics and Wald statistics. Additionally, the Koenker statistics were also statistically significant, suggesting nonstationarity or heterogeneity in the relationship between the outcome and the explanatory variables across the study areas. Therefore, local models were employed because they assume that the relationship between the independent variables and the dependent variable has spatial heterogeneity (as confirmed by Koenker statistics). We did not observe any multicollinearity issues among the explanatory variables since all the variables had a VIF of less than 7.5. The coefficient estimates displayed a combination of positive and negative values and the most influential variable. Specifically, the proportion of cesarean delivery emerged as the most influential variable, followed by the proportion of no antenatal care visit. The statistically significant variables of the EIBF included the proportion of participants who were Orthodox religion followers, the proportion of participants with a parity of 1–2, the proportion of participants with no antenatal care visit, and the proportion of participants with a cesarean delivery. (Table 3)

**Table 3:** The ordinary least square analysis (OLS) result of EIBF and EBF, 2019 EDHS

Variable	Coefficient	Robust probability	VIF	Coefficient	Robust probability	VIF
	**EIBF**			**EBF**		
Intercept	1.016	0.000000*	---	0.322	0.122	---
Proportion of child age 0-1 months				0.193	0.011333*	1.09
Proportion of rural residence	0.039	0.305	2.22	0.015	0.853	2.24
Proportion of maternal age 15-19	-0.166	0.175	1.23	-0.027	0.83	1.33
Proportion of male sex of the child	0.01	0.877	1.1	0.054	0.505	1.18
Proportion of poor wealth index	0.008	0.879	3.05	-0.003	0.972	2.06
Proportion of orthodox religion	-0.088	0.007025*	1.35	0.119	0.058	1.19
Proportion of maternal illiteracy	-0.011	0.876	3.49	0.165	0.118	2.69
Proportion of uneducated mother	0.005	0.936	3.98	-0.217	0.045715*	2.59
Proportion of home delivery	-0.099	0.143	3.79	-0.008	0.913	1.98
Proportion of parity of 1-2	-0.129	0.046677*	1.68	-0.017	0.851	1.65
Proportion of married mothers	-0.166	0.296	1.15	0.168	0.322	1.12
Proportion of no antenatal care visit	-0.189	0.030383*	2.97	-0.097	0.193	1.3
Proportion of cesarean delivery	-0.308	0.015897*	1.53	-0.205	0.162	1.29
Proportion of postnatal care service	0.006	0.947	1.16	-0.093	0.411	1.11
Proportion of exposure to media	0.058	0.333	3.43	0.011	0.906	2.4
**Ordinary least square regression diagnostics**						
	**EIBF**	**EBF**			**EIBF**	**EBF**
Number of observations	304	240	Adjusted R-squared		0.1	0.039
Joint F-statistics	3.396	1.65	Prob(> F), (6,296) degree of freedom		0.000042*	0.0633
Joint Wald statistics	53.65	19.03	Prob (> chi-squared), (6) degree of freedom		0.000001*	0.212156
Koenker (BP) statistics	39.82	15.28	Prob (> chi-squared), (6) degree of freedom		0.000272*	0.431266
Jarque–Bera	11.88	8.57	Prob (> chi-squared), (2) degree of freedom		0.002631*	0.013742*

On the contrary, the model for EBF did not yield statistically significant results, as indicated by the Joint F-statistics and Wald statistics, which were not significant. Furthermore, the Koenker statistics was not statistically significant suggesting stationarity or homogeneity in the relationship between the outcome and the explanatory variables across the study area. As a result, we were unable to apply local models for EBF. (Table 3)

#### Model comparison (global and local models)

Due to the presence of spatial non stationarity in the relationship between EIBF and the independent variables, OLS regression was not adequate for accurately describing the underlying relationship. Therefore, we employed a spatially nonstationary local modeling approaches, namely, GWR and MGWR, using the same set of predictors utilized in the global model. The local models exhibited better model performance than did the global model. The adjusted R^2^ increased from 0.100 (OLS) to 0.258 (GWR) and 0.355 (MGWR). The residual sum of squares, which shows unexplained variations, was high in the OLS model (261.1) but decreased to 194.7 (GWR) to 159.4 (MGWR). Additionally, the AICc decreased from 850.3 in OLS to 826.4 in GWR and 809.8 in MGWR. GWR outperformed OLS, while MGWR outperformed GWR. This is because MGWR allows for covariate-specific bandwidths instead of relying on a single average bandwidth, leading to improved model performance. (Table 4)

**Table 4:** Comparison of goodness-of-fit measures between the global and local models

Model comparison parameter	Adj-*R*^2^	RSS	AICc
Model			
OLS	0.100	261.05	850.3
GWR	0.258	194.7	826.4
MGWR	0.355	159.4	809.8

The GWR model utilized a single bandwidth of 237, which means that 237 nearest neighbors were considered to inform the construction of parameter estimates at each local regression point. On the other hand, the MGWR computes an optimum bandwidth for each variable. The presence of multiple bandwidths in MGWR allowed the model to account for an optimal number of neighbors to estimate each parameter, which allowed better predictions of the dependent variable. The bandwidths range from 67 to 300. Specifically, the bandwidths for the significant variables were 290 for the proportion of mothers who are orthodox religion followers, 67 for the proportion of mothers with a parity of 1–2, 71 for the proportion of mothers with no antenatal care visit, and 159 for the proportion of mothers with cesarean delivery. (Table 5)

**Table 5:** Summary of the GWR and MGWR model results for predictors of EIBF in Ethiopia, 2019 EDHS

Variable	Mean	STD	Min	Median	Max	Bandwidth
**GWR model**						
Intercept	0.031	0.059	-0.088	0.017	0.136	237
Proportion of rural resident	0.100	0.117	-0.186	0.111	0.409	237
Proportion of maternal age 15–19	-0.078	0.076	-0.187	-0.094	0.073	237
Proportion of male sex of the child	0.012	0.080	-0.158	0.031	0.157	237
Proportion of poor wealth index	0.046	0.081	-0.160	0.031	0.228	237
Proportion of orthodox religion	-0.169	0.091	-0.438	-0.142	-0.029	237
Proportion of maternal illiteracy	-0.013	0.081	-0.148	-0.028	0.175	237
Proportion of uneducated mother	0.053	0.041	-0.047	0.044	0.156	237
Proportion of home delivery	-0.100	0.156	-0.361	-0.087	0.201	237
Proportion of parity of 1–2	-0.033	0.115	-0.208	-0.079	0.216	237
Proportion of married mothers	-0.039	0.130	-0.253	0.009	0.166	237
Proportion of no antenatal care visit	-0.247	0.153	-0.516	-0.269	0.032	237
Proportion of cesarean delivery	-0.234	0.091	-0.388	-0.240	-0.087	237
Proportion of postnatal care service	-0.056	0.100	-0.197	-0.107	0.126	237
Proportion of exposure to media	0.169	0.080	0.054	0.157	0.456	237
**MGWR model**						
Intercept	0.021	0.022	-0.010	0.020	0.061	300
Proportion of rural resident	0.188	0.158	-0.196	0.214	0.594	97
Proportion of maternal age 15–19	-0.053	0.101	-0.201	-0.081	0.144	210
Proportion of male sex of the child	-0.026	0.108	-0.319	-0.010	0.147	130
Proportion of poor wealth index	0.082	0.016	0.049	0.087	0.111	302
Proportion of orthodox religion	-0.211	0.039	-0.296	-0.201	-0.156	290
Proportion of maternal illiteracy	-0.072	0.008	-0.092	-0.070	-0.057	302
Proportion of uneducated mother	0.111	0.008	0.085	0.115	0.122	302
Proportion of home delivery	-0.109	0.020	-0.144	-0.106	-0.062	302
Proportion of parity of 1–2	-0.026	0.246	-0.475	-0.109	0.482	67
Proportion of married mothers	-0.024	0.173	-0.290	0.074	0.226	191
Proportion of no antenatal care visit	-0.301	0.188	-0.666	-0.372	0.191	71
Proportion of cesarean delivery	-0.208	0.098	-0.420	-0.162	-0.105	159
Proportion of postnatal care service	-0.066	0.134	-0.254	-0.127	0.196	174
Proportion of exposure to media	0.201	0.003	0.192	0.202	0.208	302

#### Mapping parameter coefficients

**Fig. 4:**
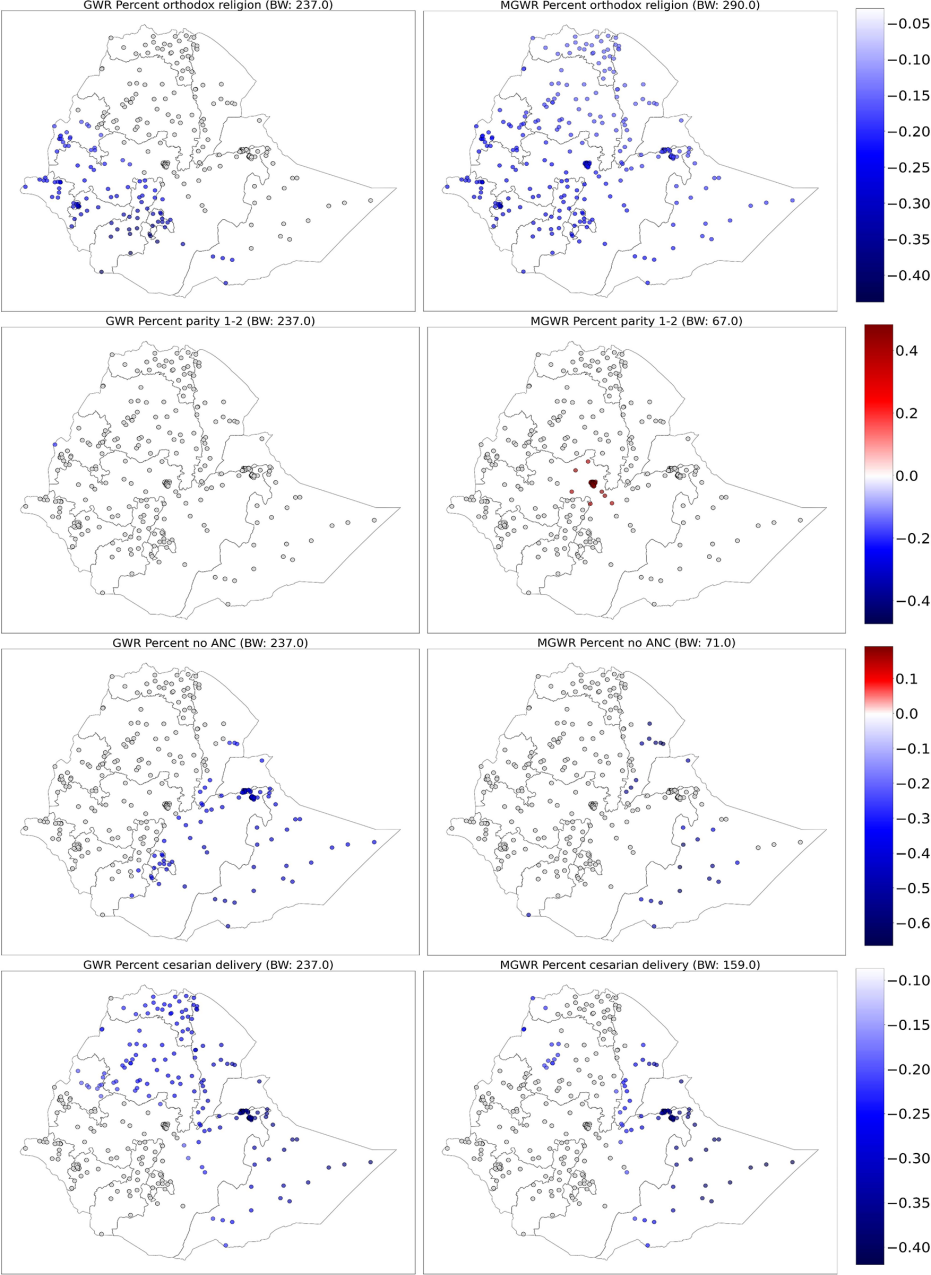
GWR (left) and MGWR (right) parameter estimates for the proportion of being orthodox religion follower, parity of 1–2, no antenatal care visit and cesarean delivery to show local patterns of spatial heterogeneity. Grey dots are not statistically different from zero

The coefficients of GWR (Fig. 4, left) and MGWR (Fig. 4, right) for EIBF were mapped for the intercept and statistically significant variables. The local intercept is interpreted as the value of the dependent variable that would be expected if every location had exactly the same average value of each independent variable. Based on the mapping of the intercept coefficients, the local intercept in the local models is interpreted as the level of EIBF that would be expected in each region holding all covariates constant. The intercepts in both the GWR and MGWR were not statistically different from zero because they had no statistically nonzero parameter estimates. (Supplementary Fig. 3)

The coefficient estimates of being the orthodox religion follower have a negative association with EIBF. Compared to the GWR, this variable displays little spatial heterogeneity in MGWR model as it has a negative association with EIBF all over the country. Regardless of location, the MGWR results support the trend that living in a neighborhood with a higher proportion of orthodox religion is linked with lower EIBF throughout the country. The MGWR also displays the coefficient estimates of the variable para 1–2, which shows a positive association with EIBF in Addis Ababa and parts of central Oromia. This finding implies that mothers who have 1 or 2 children are more likely to have EIBF in Addis Ababa and parts of central Oromia. However, the GWR estimate does not have a nonzero parameter estimate. (Fig. 4)

The MGWR reveals that parameter estimates for no antenatal care visits (absence of ANC) tend to have a negative association with EIBF in pastoralist regions of Ethiopia (central and southern Afar and central, western and southwestern Somali region). This finding indicates that mothers who did not have ANC follow-up tend to have a lower chance of engaging in EIBF in central and southern Afar and central, western and southwestern Somali regions. The GWR estimates also showed spatial heterogeneity. The coefficient estimates of the variable cesarean delivery was manifested to have a negative association with EIBF. The MGWR model showed that mothers who deliver their baby by cesarean delivery tend to have a lower chance of engaging in EIBF in parts of western Tigray, western Amhara, central and southern Afar, eastern Oromia, Harari, Dire Dawa, and most parts of the Somali region. The GWR estimates also showed spatial heterogeneity. (Fig. 4)

## Discussion

It is recommended that all newborns start breastfeeding immediately after birth, and exclusive breastfeeding for the first six months of life provides balanced nutrition and prevents child morbidity and mortality. This study examined the overall temporal trend and spatial trend as well as the projection of early initiation and exclusive breastfeeding in Ethiopia. Additionally, spatial modeling was used to explore the spatial predictors of the observed geographical variation based on the EDHS data collected in 2011, 2016 and 2019.

Between 2011 and 2019, the magnitude of the EIBF increased from 51.8% in 2011 to 71.9% in 2019; this value is considered good according to the WHO classification, where EIBF is classified as poor (0–29%), fair (30–49%), good (50–89%) or very good (90–100%) [57]. The magnitude of EBF also increased from 52.7% in 2011 to 58.9% in 2019, which was categorized as good based on the WHO classification. Similarly, previous studies reported that the prevalence of EIBF increased from 47.4% in 2000 to 66.2% in 2011 and that of EBF increased from 54.5% in 2000 to 59.9% in 2016 [31–34]. Overall, in low- and middle-income countries, 39% of children were breastfed within one hour of birth, with wide variation by region [58]. The prevalence of EIBF in Ethiopia in 2019 was greater than that in lower- and lower-middle-income countries, including Sudan (69%), Ghana (55.1%), the Economic Community of West African States (ECOWAS) (43%), India (41.5%) and Bangladesh (51.2%) [59–63]. The total prevalence of EBF among infants under six months of age in Sub-Saharan Africa was 37% [64], which is lower than the prevalence in Ethiopia. The increase in the prevalence of EIBF and EBF in Ethiopia can be attributed to government efforts, such as the national strategy for infant and young child feeding in the country and the implementation of strategies, including health extension programs [65]. However, despite the improvements in the prevalence of EIBF and EBF in Ethiopia, the country lags far from achieving its targets set by the health sector development program [30].

The national prevalence of EIBF and EBF varies by administrative region across the country. For instance, in 2019, the prevalence of EIBF ranged from 57.3% in the Somali region to 81.4% in the Oromia region. Similarly, the percentage of EBF ranged from 42.2% in Dire Dawa to 82.9% in Benishangul. This variation was confirmed by Moran’s I statistics, which revealed significant geographical variation in EIBF and EBF in the three survey years throughout the country. This findings highlight the role of geography in determining the variation in EIBF and EBF in Ethiopia.

As identified by the hotspot analysis, significant clusters of cold spots of the EIBF, where low numbers of cases of EIBF were surrounded by low numbers of cases, were consistently observed in the three survey years in the Tigray and Amhara regions. This can be explained by the repetitive occurrence of drought in the northern parts of the country, which contributes to poor breastfeeding practices. The political instabilities in these regions might also disturb the implementation of maternal and child health programs [31]. The significant clusters of cold spots in EBF were more or less consistent in the three waves of the EDHS observed in the Afar and Somali regions. This is supported by a study in Ethiopia in which pastoralist regions were less likely to exclusively breastfeed [66]. This is because their mobile lifestyle provides them with a weak healthcare system, which prevents them from understanding the importance of exclusive breastfeeding. Hence, they might initiate cow milk earlier [67].

The spatial scan analysis of EIBF revealed that in the 2011 EDHS, the most likely primary SaTScan clusters were identified in DireDawa and Harari. In 2016, EDHS those clusters were identified in Dire Dawa, Harari, eastern Oromia and the northern Somali region. Children in this spatial window had a better chance of having EIBF than did those outside the window. A previous study [68] indicated that mothers from the Oromia, Harari and Dire Dawa regions were more likely to experience EIBF. Regarding EBF, a significant primary SaTScan cluster was detected only in the 2016 EDHS in the Tigray and Amhara regions. Children in this spatial window had a better chance of EBF than did those outside the window. It has been shown that other regions have lower odds of EBF as compared to the larger central regions (including Tigray and Amhara) [34]. This spatial inequality may be related to most residents of the larger central regions being rural dwellers, and mothers residing in rural areas are more likely to exclusively breastfeed, which can be explained by differences in maternal employment [69, 70].

In this research, the MGWR model overcame the limitations of the OLS and GWR models and resulted in a well-fitted model of EIBF among children aged 0–23 months. The key findings obtained from this study were variables that impact EIBF and vary geographically. The significant predictors obtained were orthodox religion, parity of 1–2, absence of antenatal care visits, and cesarean delivery. Despite the regional location, the MGWR results showed that neighborhoods with a higher proportion of orthodox religion followers had lower rates of EIBF among children younger than 24 months in Ethiopia. This is supported by a similar study in which GWR was utilized in Ethiopia [36]. The parity of 1–2 have more regional and local relationships with EIBF. Parity of 1–2 children has a significant positive relation with EIBF in Addis Ababa and parts of central Oromia. This can be explained by the fact that mothers who are multipara or grand multipara have better prior breastfeeding experience and fewer breastfeeding problems, unlike primiparous women, who usually have delays from delivery to their first breastfeeding attempt. The more experience the mother has, the more likely the infant is to be put to the breast within one hour of delivery [71–73].

Having no antenatal care follow-up had a significant negative association with EIBF in central and southern Afar and central, western and southwestern Somali region or pastoralist regions of Ethiopia. This could be due to the counseling and support services provided for newborn feeding practices that mothers obtain when attending ANC visits. ANC is also a means of enhancing mothers’ understanding of the advantages of EIBF by overcoming cultural obstacles related to infant feeding practices [74, 75]. Furthermore, mothers from pastoral regions in Ethiopia utilize health facility services at low levels as they move long distances with their livestock following seasonal movements [76].

Children who were delivered by cesarean section had lower rates of EIBF in parts of the western Tigray, western Amhara, central and southern Afar, eastern Oromia, Harari, Dire Dawa, and most parts of the Somali region. This finding is consistent with a study conducted in Ethiopia utilizing GWR [36]. Several studies have shown that cesarean delivery plays a role in delaying breastfeeding initiation. A possible explanation might be that separating newborns from their mothers after CS leads to insufficient milk production, reduced time spent at the mother’s breast, decreased neonatal interest in breastfeeding and worsening of the wellbeing and psychology of the mother, which might contribute to delays in breastfeeding initiation [77–80].

### Strength and limitations

This study has limitations that must be considered when the results are interpreted. As the DHS data are cross-sectional and only capture a snapshot of information at a particular moment, we were unable to show the cause‒effect relationship between the outcome variable and covariates being examined. Participant responses are also prone to recall bias, as the survey used a 24-h recall method for measuring EBF, but we conducted the analysis on the youngest child who lived with the mother to reduce the effect of bias. Despite these limitations, the study has several strengths. The study is conducted based on weighted, nationally representative data. Appropriate spatial statistical methods were also used to geographically target interventions. GWR and MGWR were conducted on the basis of the latest DHS data from 2019 to identify spatial predictors of EIBF among children under the age of 24 months.

## Conclusion

Three consecutive national surveys were used (EDHS 2011, 2016 and 2019), and the trends in EIBF and EBF have shown an increase over time despite still being below the national target. EIBF and EBF varied geographically across regions, and the distributions were nonrandom throughout the three surveys. Therefore, public health programs that improve maternal healthcare service utilization and maternal education should target the cold spot areas of EIBF and EBF in the country. This study utilized a spatial modeling framework to explore the relationship between EIBF and a set of covariates in Ethiopia. Global and local models were applied and compared to explain the spatial heterogeneity, and the MGWR provided the best overall fit. The single-bandwidth assumption of GWR was relaxed by MGWR, in which each covariate had its own bandwidth. The explanatory variables that had a significant influence on the spatial variation in EIBF in Ethiopia were being a follower of the orthodox religion, having a parity of 1–2, not visiting antenatal care, and a cesarean delivery. To address these barriers, the government should integrate facility-level services at the community level to achieve optimal breastfeeding. Mother-to-mother support groups can be utilized to sustain gains in breastfeeding; specifically, multiparous mothers can share their experience with nulliparous and primiparous mothers. To prevent delayed initiation of breastfeeding and early discontinuation of EBF, it is important to address postpartum exhaustion, discomfort, pain and any issues related to cesarean delivery. Strategies and guidance for breastfeeding should be promoted for mothers who experience CS delivery. Antenatal care service utilization must also be encouraged by creating awareness, ensuring service accessibility and availability and improving quality of care. For pastoralist communities, maternal health care services should be suited to their mobile lifestyle, limited access to health care facilities and cultural considerations. The mobile-clinic approach can reach pastoralist communities in different locations by providing health care services directly.

### Electronic supplementary material

Below is the link to the electronic supplementary material.

Supplementary Material 1

## Data Availability

The data are available from http://www.dhsprogram.com.

## Abbreviations

AIC: Akaike information criterion
CS: Cesarean Section
EAs: Enumeration areas
EBF: Exclusive Breastfeeding
EDHS: Ethiopian Demographic and Health Survey
EIBF: Early Initiation of Breastfeeding
GWR: Geographically Weighted Regression
LMICs: low- and middle-income countries
MGWR: Multiscale Geographically Weighted Regression
OLS: Ordinary Least Square
RSS: Residual Sum of Squares
SDG: Sustainable Development Goal
SNNP: Southern Nations Nationalities Peoples
SSA: Sub-Saharan Africa
WHO: World Health Organization
