# Supplementary material for: Geospatial determinants and spatio-temporal variation of early initiation of breastfeeding and exclusive breastfeeding in Ethiopia from 2011 to 2019, a multiscale geographically weighted regression analysis
Source: BMC Public Health. 2024 Jul 27;24:2011. doi: 10.1186/s12889-024-19552-0 (PMC11282616; doi:10.1186/s12889-024-19552-0)
Supplement: Supplementary file 1 — Supplementary Material 1 [file 12889_2024_19552_MOESM1_ESM.docx]

**Geospatial determinants and spatio-temporal variation of early initiation of breastfeeding and exclusive breastfeeding in Ethiopia from 2011-2019, a multiscale geographically weighted regression analysis**

**Supplementary figures**


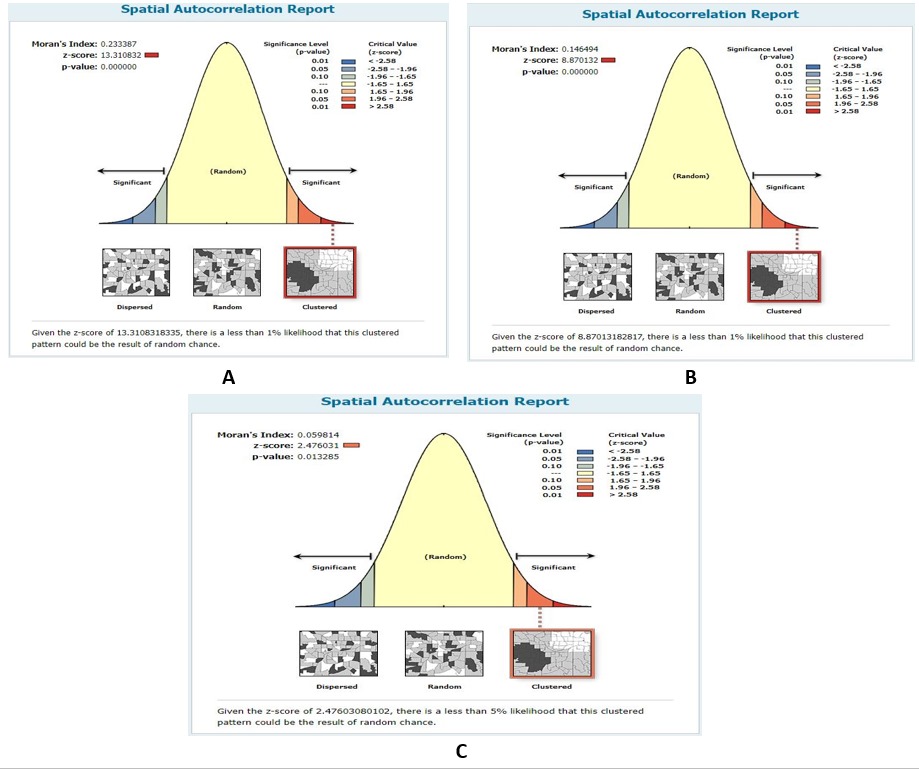


Supplementary Figure 1: The global spatial autocorrelation analysis of EIBF among adolescents aged 0-23 months in Ethiopia; 2011(A), 2016(B), and 2019(C)


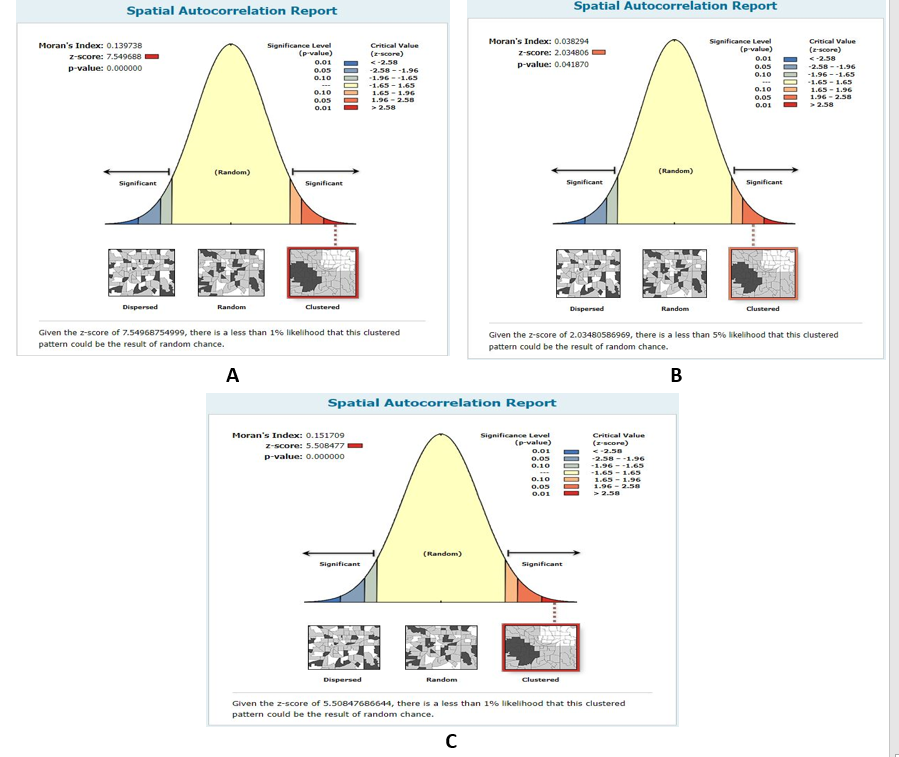


Supplementary Figure 2: The global spatial autocorrelation analysis of EBF among adolescents aged 0-5 months in Ethiopia; 2011(A), 2016(B), and 2019(C)


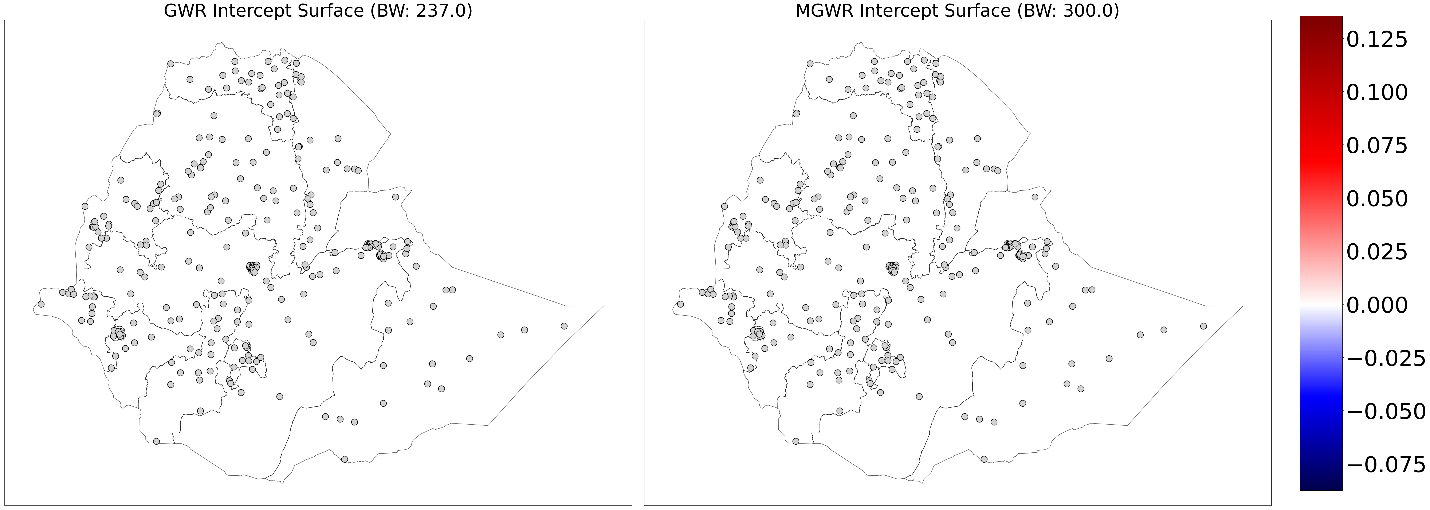


Supplementary Figure 3: GWR (left) and MGWR (right) parameter estimates for the intercept to show local patterns of spatial heterogeneity. Grey dots are not statistically different from zero
